# Assessment of the diagnostic sensitivity and specificity of an indirect ELISA kit for the diagnosis of *Brucella ovis* infection in rams

**DOI:** 10.1186/1746-6148-8-68

**Published:** 2012-07-09

**Authors:** Anne Praud, Jean-Luc Champion, Yannick Corde, Antoine Drapeau, Laurence Meyer, Bruno Garin-Bastuji

**Affiliations:** 1 National Veterinary School of Alfort (ENVA) / French Agency for Food, Environmental and Occupational Health Safety (ANSES), USC Epidemiology of Animal Infectious Diseases Unit (Epi-MAI), 94700, Maisons-Alfort, France; 2 Groupement de Défense Sanitaire des Alpes de Haute Provence, 04000, Digne les Bains, France; 3 ANSES, Animal Health Laboratory, EU/OIE/FAO Brucellosis Reference Laboratory, 94706, Maisons-Alfort, France; 4 INSERM, Centre for research in Epidemiology and Population Health (CESP), U1018, Faculté de Médecine Paris-Sud, Le Kremlin-Bicêtre; AP-HP, Hopital Bicêtre, Epidemiology and Public Health Service, Université Paris-Sud, 94276, Le Kremlin-Bicêtre, France

**Keywords:** *Brucella ovis*, Diagnostic tests, CFT, I-ELISA, Sensitivity, Specificity, Bayesian approach

## Abstract

**Background:**

*Brucella ovis* causes an infectious disease responsible for infertility and subsequent economic losses in sheep production. The standard serological test to detect *B. ovis* infection in rams is the complement fixation test (CFT), which has imperfect sensitivity and specificity in addition to technical drawbacks. Other available tests include the indirect enzyme-linked immunosorbent assays (I-ELISA) but no I-ELISA kit has been fully evaluated.

The study aimed to compare an I-ELISA kit and the standard CFT. Our study was carried out on serum samples from 4599 rams from the South of France where the disease is enzootic. A Bayesian approach was used to estimate tests characteristics (diagnostic sensitivity, Se and diagnostic specificity, Sp). The tests were then studied together in order to optimise testing strategies to detect *B. ovis*.

**Results:**

After optimising the cut-off values in order to avoid doubtful results without deteriorating the concordance between the results of the two tests, the I-ELISA appeared to be slightly more sensitive than CFT (Se _I-ELISA_ = 0.917 [0.822; 0.992], 95% Credibility Interval (CrI) compared to Se _CFT_ = 0.860 [0.740; 0.967], 95% CrI). However, CFT was slightly more specific than I-ELISA (Sp _CFT_ = 0.988 [0.947; 1.0], 95% CrI) compared to Sp _I-ELISA_ =0.952 [0.901; 1.0], 95% CrI).

The tests were then associated with two different interpretation schemes. The series association increased the specificity of screening and could be used for pre-movement testing in rams from uninfected flocks. The parallel association increased sequence sensitivity, thus appearing more suitable for eradicating the disease in infected flocks.

**Conclusions:**

The high sensitivity and acceptable specificity of this I-ELISA kit support its potential interest to avoid the limitations of CFT. The two tests could also be used together or combined with other diagnostic methods such as semen culture to improve the testing strategy. The choice of test sequence and interpretation criteria depends on the epidemiological context, screening objectives and the financial and practical constraints.

## Background

*Brucella ovis* is a Gram-negative coccobacillus. In sheep, *B. ovis* infection is responsible for a reproductive disease, often causing genital lesions such as unilateral or bilateral epididymitis in rams and, more rarely, abortion in ewes. This disease mainly spreads via venereal transmission, even though other routes of infection have been observed. Infected ewes generally clear the micro-organism from the vagina within two oestrus cycles [1], but the clearance period can extend up to three months [2]. It has also been suggested that ewes could play a role in the maintenance of the infection in flocks [3,4].

*B. ovis* infection in sheep was first reported in 1953 in Australia and New Zealand [5]. It is currently present in South and North American countries, Australia, New-Zealand, South Africa and Southern European countries [6]. In France, the number of infected flocks has increased since Rev.1 vaccination against *B. melitensis* infection was stopped in 2008.

The infection generates economic losses in infected flocks (decrease in fertility, ban on trade). These losses must be taken into account when evaluating the most suitable screening strategy. Financial losses are principally due to a drop in fertility, with recycling ewes commonly observed in an infected flock. Reproductive failure rates depend on the extent of lesions: if only one testicle is involved, conception rates may be 70%, whereas in healthy rams conception rates of 90% can be expected [7]. Estimates of the abortion rate in ewes and perinatal mortality vary from 0% to 8% in experimental studies. Furthermore, lambs born in the second and third cycle are 10–20 lbs lighter at weaning which can equate to a loss of $10 to $20 for each cycle missed [7]. *B. ovis* infection also induces indirect losses such as a shorter reproductive career, a decrease in the economic value of rams or an increase in the number of rams needed per ewe [8]. These observations emphasise the importance of developing suitable testing strategies in various control and eradication situations.

The diagnosis of *B. ovis* infection mainly depends on serological tests. The clinical detection of the disease is difficult because other bacteria, such as *Actinobacillus seminis, Histophilus ovis, Haemophilus* spp*., Corynebacterium pseudotuberculosis ovis, Chlamydophila abortus* or *B. melitensis*, may cause similar symptoms and more than 50% of *B. ovis* infected animals do not show any palpable epididymitis lesion [9]. Infected rams excrete *B. ovis* in semen intermittently, so the bacteriological examination of semen is not very sensitive [10].

As in many other parts of the world, there is currently no compulsory surveillance of the disease in EU flocks. Moreover, neither compulsory eradication programme nor compensation scheme for culling animals in infected flocks is foreseen in the EU Member States. Nevertheless, in order to avoid the contamination of non-infected areas or flocks through international or intra-community trade, rams have to undergo serological pre-movement tests [11]. Rams are also tested before their admission to artificial insemination units. On farms, diagnosis mainly relies on a clinical detection and a serological test when the palpation of testicles reveals lesions or when there is significant infertility in the flock.

Various tests are available to detect *B. ovis* antibodies in serum, such as the complement fixation test (CFT), agar gel immunodiffusion (AGID) or indirect enzyme-linked immunosorbent assay (I-ELISA) but only CFT is prescribed for international or intra-community trade ([6,12]). CFT has good sensitivity and specificity but also has some technical drawbacks such as anti-complementary activity [13], prozone phenomenon [14], incompatibility with haemolysed sera ([10,14]), serum inactivation [14] and workload [15].

Other tests such as the indirect ELISAs (I-ELISA) are available but no I-ELISA kit has been fully evaluated in previous studies. According to literature data, some I-ELISAs appear more sensitive than CFT ([12,16]), but there are differences in the contexts (various geographical areas, breeds and breeding conditions of the animals, sample sizes, tests manufacturers and cut-off values) and the statistical methods used for comparison (CFT being considered as the gold standard and estimation of relative sensitivity and specificity of I-ELISA) ([15-19]). Advantages of I-ELISA include its ease of use; it is less labour intensive than CFT and can be used to test haemolysed or anti-complementary serum samples [15].

Our study compared a commercial I-ELISA kit and standard CFT in order to evaluate its potential use as a complementary test for international trade or for diagnosis on farms, and to assess its possible use instead of CFT or in association with it to improve the detection and eradication of the disease and the protection of disease-free flocks.

## Methods

### Serum samples

The study included sera from 4,599 rams collected in the South of France, in areas where *B. ovis* infection is enzootic: 3,063 were from Pyrénées Atlantiques (subpopulation 1; individual prevalence estimated at 22% in 2006 [20]), 1,340 were from Provence-Alpes-Côte d’Azur (subpopulation 2; enzootic infection but prevalence unknown) and 196 young rams, between 12 and 14 months of age, were from a cooperative that was found to be infected, *B. ovis* being isolated in some animals (subpopulation 3). A free population used as a source of prior information on the specificity of I-ELISA included 3,792 rams selected from free artificial insemination (AI) units.

Blood samples were collected as part of the usual official screening scheme on farms (and not specifically for research) The EU recommendations about screening procedures and EU ethical guidelines and animal welfare regulations were strictly respected.

### Serological tests

CFT and I-ELISA (Chekit *B. ovis*, Idexx, France) were performed in parallel on all sera from the infected populations described above. The antigens used for both CFT and I-ELISA consisted in a hot-saline water soluble extract of the REO 198 strain prepared according to OIE recommendations [4]. The CFT antigen (ANSES, France) was standardized against the International Standard anti-*Brucella* ovis Serum (ISaBoS; AHVLA, UK) and the test performed according to OIE and EU requirements with a positivity threshold of 50 ICFTU/mL ([6,11]). I-ELISA included an anti-ruminant IgG monoclonal antibody labelled with horseradish peroxidase as conjugate, 3,3′,5,5′-Tetramethylbenzidine (TMB) as substrate and a slightly acidic detergent solution as stopping reagent. Serum samples and conjugate incubation steps were performed at 37 °C for 60 min. (± 10 min.). Substrate was incubated at room temperature for 15 min. Optical densities (450 nm) were then transformed in % values by comparing the test-sample optical density (OD) to the positive control OD, both ODs being corrected by subtracting the negative control OD. The manufacturer’s recommended cut-offs were negative if under 10%, doubtful between 10% and 50% and positive above 50%. The reproducibility of each batch used in the study was checked against the ISaBoS on two plates per batch. All tests were performed by the same two technicians at the EU/OIE/FAO Brucellosis Reference Laboratory (ANSES, Maisons-Alfort, France). The blood samples were collected in the frame of a national official surveillance program. All samples were provided to the authors’ laboratory either by the local breeders’ organizations (GDS) or by the National Laboratory for the Control of Breeding Animals without any Material Transfer Agreement. Therefore these blood samples could be freely used for the production of any additional data.

### Data analyses

A new cut-off was established (see additional details in the Results section) to eliminate doubtful results without deteriorating the concordance between the results of CFT (the officially prescribed test) and I-ELISA.

Then, each test’s sensitivity (Se) and specificity (Sp) were estimated using a Bayesian approach implemented through Markov Chain Monte Carlo algorithms. The Bayesian approach has often been used to estimate sensitivities and specificities of tests in the absence of a gold standard, whether in veterinary or human medicine ([21-23]). The main advantage of this method is to combine prior information from the literature and experts’ advice with field data.

The model used was a generalization of the Bayesian independence model to allow for dependent test outcomes. It has been applied in previous studies ([21,24-26]) and used to estimate the respective covariance of the sensitivities (γSe) and of the specificities of the two tests (γSp). Two tests are said to be independent of, given that an animal is diseased (or not), the probability of positive (or negative) outcome for the first test is the same whatever the outcome for the other test [27]. Both tests are based on the same biological process: they use the same HS antigen preparation and therefore detect mainly anti-*Brucella*-R-LPS antibodies. They are consequently expected to be conditionally dependent [24].

Prior distributions of parameters were modelled as beta distributions ([27,28]). To construct a beta prior distribution, the most probable value of the parameter (or “best guess”; θ_0_) and a “lower limit” (θ_L_*; i.e.* a value for which the experimenter is 95% sure that the parameter will be larger) were determined [27]. Table 1 provides the parameters of the beta (a,b) distribution for each parameter estimated by the model.

**Table 1:** Parameters of the Beta(a,b) distributions used as priors

**Parameter**		**Mean**	**Lower limit (95%)**	**Parameters of beta distribution**	
				**a**	**b**
**CFT**	Se*	0.9	0.8	31.5	3.5
	Sp*	0.95	0.8	7.07	0.37
**I-ELISA**	Se*	Unknown	Unknown	1	1
	Sp*	0.98	0.9	11.03	0.23
	γ Se*	Unknown	Unknown	1	1
	γ Se*	Unknown	Unknown	1	1
		Unknown	Unknown	1	1

*Se = Sensitivity; Sp = Specificity; **γ =** covariance.

Prior information on CFT combined literature and experts’ advice with field data. Only prior information from similar studies in literature was input into the model. According to previous studies, mean CFT sensitivity ranged from 88.9% to 98.7% and mean CFT specificity from 89.3% to 100%. The lowest sensitivity reported was 77% and the lowest specificity 78% ([10,14,29-33]).

Prior information on I-ELISA specificity (Sp I-ELISA) was input from a study performed on the sera from the 3,792 rams selected in officially brucellosis free AI units. As these rams were being raised in specific conditions, with little contact with environmental pathogens potentially responsible for cross-reactions, they were not representative of the population bred on farms in the South of France. They were not therefore studied with the population of 4,599 rams from the South of France but used to provide prior information on the specificity of I-ELISA (Sp I-ELISA = 99.74%).

Since little information was available in the literature on this I-ELISA sensitivity and since the corresponding studies were carried out with very different methods, in different contexts, as indicated above, beta (1,1) uninformative distributions (uniform distributions between 0 and 1) were chosen.

Prevalence in the population and covariance of the sensitivity and the specificity were unknown: beta (1,1) uninformative distributions were also used as priors for these parameters.

The statistical analysis was performed with the WinBUGS program [34]. The MCMC algorithm convergence was assessed by checking the stabilisation of the plots of iterate parameter value, after a given number of samples and by running multiple chains from dispersed starting values. Early samples (1,000 out of 50,000) were discarded as a “burn-in” period. A sensitivity analysis was also performed by making the prior distributions more diffuse in order to check that the parameter estimates were little affected by these variations [27].

After having estimated the sensitivity and the specificity of the two tests, their characteristics were evaluated when used in association [35]. With the serial association, the overall result was positive when the results were positive with both tests. With the parallel association, the overall result was positive when at least one test gave a positive result. The sensitivity and specificity of the combined tests were estimated taking into account the conditional dependence between results [24]. The sensitivity of the serial association was: Se_SERIES_ = Se_IELISA_*Se_CFT_ + γSe and its specificity was: Sp_SERIES_ = (1 - Sp_IELISA_)*(1 – Sp_CFT_) – γSp. The sensitivity of the association in parallel was: Se_PARALLEL_ = 1-((1-Se_IELISA_)*(1-Se_CFT_)) – γ Se and its specificity was: Sp_PARALLEL_ = Sp_IELISA_ * Sp_CFT_ + γ Sp; (with Se: sensitivity, Sp: specificity, γ: covariance).

## Results

A new cut-off was established for I-ELISA in order to eliminate doubtful results without affecting the concordance between the results of CFT (the officially prescribed test) and I-ELISA. A cut-off at 45% OD (see above) was the best compromise to optimise both the concordance of positive results and the concordance of negative results. With this new cut-off, the two tests gave concordant results (positive / positive or negative / negative) for 4,161 rams (92.6%).

The apparent prevalence assessed in the whole studied population by the I-ELISA kit was 25.6%. In the subpopulations 1, 2 and 3, the apparent prevalences were respectively 17.0%, 44.8% and 27.6%. Table 2 gives the results of the 4,599 tested rams.

**Table 2:** Cross-classified results in 4,599 rams from the South of France

**CFT**	I-ELISA*	**Number of rams***(proportion)*
Negative	Negative	3,335 *(72.8%)*
Negative	Positive	270 *(5.9%)*
Positive	Negative	72 *(1.6%)*
Positive	Positive	902 *(19.7%)*
**TOTAL**		4,599 (100%)

* cut-off: 45% OD.

The I-ELISA kit was slightly more sensitive than the CFT (Se I-ELISA = 0.917 [0.822; 0.992], 95% Credibility Interval (CrI) whereas Se CFT = 0.860 [0.740; 0.967], 95% CrI). In contrast, the CFT was somewhat more specific than the I-ELISA (Sp CFT = 0.988 [0.947; 1.0], 95% CrI whereas Sp I-ELISA = 0.952 [0.901; 1.0], 95% CrI).

Prevalence in the studied population of 4,599 from the South of France was evaluated at 0.238 ([0.187; 0.284], 95% CrI). The sensitivity covariance was 0.0241 ([−0.00773; 0.0949], 95% CrI) and the specificity covariance was 0.00521 ([−4.61 10^-7^; 0.0361], 95% CrI).

These two tests could also be associated to improve diagnosis reliability. Two interpretation schemes were studied: a serial association and a parallel association. The estimations of sensitivity and specificity of each sequence were based on the individual sensitivities and specificities described above.

The diagnostic sensitivity of the association in series was Se_SERIES_ = 0.812 [0.699; 0.980] (95% CrI) and its specificity was Sp_SERIES_ = 0.995 [0.994; 0.996] (95% CrI). The diagnostic sensitivity of the parallel association was Se_PARALLEL_ = 0.964 ([0.902; 0.983], 95% CrI) and its specificity was Sp_PARALLEL_ = 0.946 ([0.854; 1.0], 95% CrI).

## Discussion

The estimation of the mean prevalence in the sample (24%) confirmed the value assessed previously in French areas in which *B. ovis* infection is enzootic [20].

The estimation of the diagnostic sensitivity and specificity of a test should ideally be derived from testing a statistically relevant panel of animals. The history of these animals and their infection status should be known. The panel should be representative of the region where the test is to be used [36]. As mentioned above, there is currently no regulatory program in the EU for eradicating the disease or surveying the absence of *B. ovis* infection (*i.e*. with an appropriate definition of a *B. ovis*-free status), except in AI units. In these units, animals are kept in specific conditions that could lead to an overestimation of tests specificity. Designing an adequate sampling of a sufficient number of free animals (from free flocks) is therefore very difficult. Moreover, the selection of an appropriate gold-standard-infected population (*i.e.* culture-positive animals) is extremely cumbersome and requires a very important budget Furthermore, *Brucella*-culture should be performed in conditions providing optimised sensitivity, with a corresponding increase in costs, in order to prevent any consecutive overestimation of the diagnostic sensitivity of the serological tests under study. The technical and financial constraints highlighted above justify the use of the Bayesian approach to estimate the characteristics of serological tests for the diagnosis of *B. ovis* infection when the infection status of each animal of the studied population is unknown.

The outcome of our study is consistent with previous results available in the literature. The CFT has a good diagnostic sensitivity and an acceptable specificity but has some technical disadvantages. The EU/OIE/FAO Reference laboratory estimated that this I-ELISA commercial kit was cheaper than the CFT (in-house produced antigen) when the cost was calculated for a daily analysis of 90 serum samples, mainly due to lower manpower costs (data not shown). Moreover, the evaluated I-ELISA is standardised and easier and quicker to perform. Altogether, these advantages allow the analysis of more samples by a single technician in a working day than the CFT. Moreover, the I-ELISA can be automated to speed up the process. Furthermore, haemolysed and anti-complementary sera do not affect I-ELISA performance ([15,32]). These advantages may further reduce the cost of this I-ELISA compared to the CFT.

In a screening situation, one important issue is the interpretation of doubtful results obtained with the I-ELISA. We established a cut-off different from that recommended by the manufacturer to avoid doubtful results without decreasing the concordance between the results of the two tests. Nevertheless, it is possible to adjust this cut-off according to the epidemiological context. When the test is used for international or intra-community trade, false positive results due to a lack of specificity could hinder trade and raise economic issues in brucellosis free contexts. Exported rams should come exclusively from uninfected farms (*i.e.* showing only negative results to regular tests), so the positive predictive value of the test results is low. For this reason, a high cut-off value could be used (for instance 60% OD). In contrast, when the test is used to diagnose *B. ovis* infection in infected areas or to confirm the infection in rams with clinical lesions with the aim of eradicating the disease, false negative results might slow down eradication and, in particular conditions, it could be acceptable to cull seropositive but uninfected rams. Accordingly, in a context of high prevalence, a low cut-off value could therefore be used (for example 30% OD).

The imperfect diagnostic performance of both tests could justify their use in association [35]. With the serial association, the overall result is positive when the results are positive with both tests. This interpretation scheme increases the diagnostic specificity of screening and could therefore be recommended for pre-movement testing in a brucellosis free context, in order to avoid false positive results due to the relative lack of diagnostic specificity of this I-ELISA. With the parallel association, the overall result is positive when at least one test gives a positive result.

The parallel association increases the sensitivity of the screening. Accordingly, it could be recommended in a context of suspected infection with the aim of accelerating the eradication of the disease. In this context, many sera may have to be tested in a flock and the rapidity of the process influences the success eradication operations.

The following situations would be suitable for application of screening strategies using the I-ELISA kit:

Rams for export must come from brucellosis free flocks. In this case, the animals could be tested with I-ELISA alone. If all results were negative, rams could be qualified for export. Rams from flocks showing positive results to I-ELISA should not be exported and should be subjected to further investigations (clinical examination, CFT, semen culture or PCR for instance) to confirm or rule out the infection.

In infected flocks in which *B. ovis* has already been identified, the testing regime should be focused on a quickly eradication of the disease. In this case, all animals (including females) should be submitted to I-ELISA and CFT in parallel. Animals with positive results to at least one of the two tests should be culled and replaced by virgin animals from flocks free of *B. ovis* infection. The remaining animals resulting negative to both tests could be kept in the flock but should neither be sold nor exported. Further tests should be carried out at 45- to 60-days intervals until all infected animals have been eliminated (*i.e.* after the entire animal population has been tested negative twice). This strategy raises financial issues in infected flocks with a high prevalence of the disease, because many animals would be culled.

In all flocks, animals should be submitted to a clinical examination two months before the breeding season. Rams with clinical signs of epididymitis should be banned from reproduction and subjected to an I-ELISA test. Rams with positive results to I-ELISA should undergo further investigations (semen culture) to confirm the presence of *Brucella ovis*.

## Conclusions

Our study used a Bayesian approach to evaluate the diagnostic performance of a commercially available I-ELISA kit to detect *B. ovis* infection in sheep. We showed that this I-ELISA kit was slightly more sensitive than the CFT, but somewhat less specific. The high sensitivity and acceptable specificity of the I-ELISA kit support its potential interest for diagnosing *B. ovis* infection in sheep. This I-ELISA kit has certain advantages over CFT and could be used alone or as a first test in associated testing regimes. The interpretation of the results and the testing sequences should be chosen depending on screening objectives, the disease’s prevalence in the target population and economic and practical constraints.

## Abbreviations

CFT: Complement fixation test; I-ELISA: Indirect enzyme-linked immunosorbent assay; AI: Artificial insemination; ISaBoS: International standard anti-Brucella ovis serum; Se: Sensitivity; Sp: Specificity; γ: Covariance; CrI: Credibility interval.

## Competing interests

The authors declare that they have no competing interests.

## Authors' contributions

JLC and BGB organized the sample collection. BGB conceived and designed the testing study and directed the test performance. YC and AD performed all the immunological analyses and helped design the testing study. AP conceived the model, performed data analyses and drafted the manuscript. AP, JLC, LM, YC, AD and BGB contributed to the conception and design of the whole study, revised the manuscript and approved the final manuscript.

## Authors’ information

AP: DVM, Teacher and researcher in National Veterinary School of Maisons-Alfort. JLC: DVM, Veterinary practitioner, Veterinary Advisor of the *Groupement de Défense Sanitaire des Alpes de Haute Provence*. LM: MD, PhD, Professor of Public Health at Paris Medical University. YC and AD: BSc. (Biol.). BGB: DVM, PhD, Senior Research Director, Head of the EU/OIE/FAO Brucellosis Reference Laboratory (ANSES).
